# Constraints on exotic spin-velocity-dependent interactions

**DOI:** 10.1038/s41467-022-34924-z

**Published:** 2022-11-30

**Authors:** Kai Wei, Wei Ji, Changbo Fu, Arne Wickenbrock, Victor V. Flambaum, Jiancheng Fang, Dmitry Budker

**Affiliations:** 1 School of Instrumentation Science and Opto-electronics Engineering, Beihang University, 100191 Beijing, China; 2 Hangzhou Extremely Weak Magnetic Field Major Science and Technology Infrastructure Research Institute, 310051 Hangzhou, China; 3 Hangzhou Innovation Institute, Beihang University, 310051 Hangzhou, China; 4 Helmholtz-Institut, GSI Helmholtzzentrum für Schwerionenforschung, Mainz, 55128 Germany; 5 Johannes Gutenberg-Universität Mainz, Mainz, 55128 Germany; 6 Key Lab of Nuclear Physics & Ion-beam Application (MoE), Institute of Modern Physics, Fudan University, 200433 Shanghai, China; 7 School of Physics, University of New South Wales, Sydney, NSW 2052 Australia; 8 Department of Physics, University of California, Berkeley, CA 94720-7300 USA

**Keywords:** Particle physics, Quantum metrology

## Abstract

Experimental searches for exotic spin-dependent forces are attracting a lot of attention because they allow to test theoretical extensions to the standard model. Here, we report an experimental search for possible exotic spin-dependent force, specifically spin-and-velocity-dependent forces, by using a K-Rb-^21^Ne co-magnetometer and a tungsten ring featuring a high nucleon density. Taking advantage of the high sensitivity of the co-magnetometer, the pseudomagnetic field from this exotic force is measured to be ≤7 aT. This sets limits on coupling constants for the neutron-nucleon and proton-nucleon interactions in the range of ≥0.1 m (mediator boson mass ≤2 *μ*eV). The coupling constant limits are established to be $|{g}_{V}^{n}|\,\le \,8.2\times 1{0}^{-11}$ and $|{g}_{V}^{p}|\,\le \,3.7\times 1{0}^{-10}$, which are more than one order of magnitude tighter than astronomical and cosmological limits on the coupling between the new gauge boson such as Z’ and standard model particles.

## Abstract (web summary)

Exotic spin-dependent force are among the possible extensions of the Standard Model that can be probed by precision measurements. Here, the authors use a spin-exchange-relaxation free (SERF) K-Rb-21Ne comagnetometer to improve limits on spin- and velocity dependent forces.

## Introduction

Precision measurements are powerful tools to find new physics beyond the Standard Model. For example, the discrepancies revealed by the precision measurements of the muon anomalous magnetic moment^1,2^ and the proton radius^3,4^ have been analyzed as possible indications of “new” physics. Light particles, including the spin-1 boson Z’ and spin-0 Axion Like Particles (ALPs) were proposed to resolve these discrepancies^5,6^, and are also promising candidates for dark matter^7–9^. If these particles exist, they mediate new long-range spin-dependent forces^10,11^, and could be discovered in precision measurements.

Many experiments are conducted to search for long-range forces. Typical approaches include torsion pendulums^12^, torsional oscillator^13^, atomic magnetometers^14–17^, nuclear magnetic resonance^18–21^, nitrogen-vacancy (NV) centers in diamond^22^, magnetic microscopes^23^, polarized neutron experiments^24–26^, and measurements of atomic and molecular electric dipole moments^27^.

Mathematically, forces between two particles, which depend on velocity, spin, and distance, can be broken down into 15 terms of spin-dependent forces^10,11,28^, which provides a guide on how to search them experimentally. Among the 15 terms, the spin-and-velocity-dependent (SVD) terms have received extensive attention in recent years^16,19,22,23,29^. Considering that these forces are mediated by Z’, the corresponding Lagrangian can be expressed as^11^

1 $${{{{{{{{\mathcal{L}}}}}}}}}_{Z{\prime} }={Z}_{\mu }^{{\prime} }\mathop{\sum}\limits_{\psi }\bar{\psi }{\gamma }^{\mu }\left({g}_{V}+{\gamma }^{5}{g}_{A}\right)\psi,$$

where *ψ* is the fermion field, *γ*^5^ and *γ*^*μ*^ are Dirac matrices, and *g*_*A*_ and *g*_*V*_ are axial and vector coupling constants. One of the SVD potentials, *V*_4+5_, as being noted in Ref. [10], can be derived from this Lagrangian^10,19^,

2 $${V}_{4+5}=\frac{-{f}_{4+5}{\hslash }^{2}}{8\pi mc}\left[\left(\hat{{{{{{{{\boldsymbol{\sigma }}}}}}}}}\cdot ({{{{{{{\boldsymbol{v}}}}}}}}\times \hat{{{{{{{{\bf{r}}}}}}}}})\right.\right]\left(\frac{1}{\lambda r}+\frac{1}{{r}^{2}}\right){{{{{{\rm{e}}}}}}}^{-r/\lambda },$$

where *f*_4+5_ = − (*g*_*A*_ *g*_*A*_ + 3*g*_*V*_ *g*_*V*_)/2 is a dimensionless coupling factor, *ℏ* is the reduced Planck constant, *c* is the speed of light, *λ* is the force range, **r** and **v** are the relative distance and velocity between the two particles, $\hat{\sigma }$ is the spin of one fermion and *m* is its mass.

One can write *V*_4+5_ as *V*_4+5_ = − ***μ*** ⋅ **B**_psd_, where **B**_psd_ is the pseudomagnetic field, and *μ* is the magnetic moment of the particle. Using bulk test material, the total pseudomagnetic field can be computed by integration over the volume of the material,

3 $${{{{{{{{\bf{B}}}}}}}}}_{{{{{{{{\rm{psd}}}}}}}}}\equiv \frac{{f}_{4+5 }{\hslash }^{2}}{8\pi mc\mu }\int{\rho }_{N}({{{{{{{\bf{r}}}}}}}})({{{{{{{\boldsymbol{v}}}}}}}}\times \hat{{{{{{{{\bf{r}}}}}}}}})\left(\frac{1}{\lambda r}+\frac{1}{{r}^{2}}\right){{{{{{\rm{e}}}}}}}^{-r/\lambda }{{{{{{{\rm{d}}}}}}}}{{{{{{{\bf{r}}}}}}}},$$

where *ρ*_*N*_(**r**) is the mass-source nucleon density at location **r** with the sensor chosen as the origin, where the “N” notes the average nucleon contribution to **B**_psd_ from the mass-source nucleons which is a linear combination of the protons and neutrons in the material (for example, 74 protons and 110 neutrons for tungsten gives 74/184 proton and 110/184 neutron interaction constants). Accordingly, the exotic force decays exponentially with the relative distance and it is beneficial to use high-density test materials. In fact, non-magnetic materials, such as silica (nucleon density 1.33 × 10^24^ cm^−3^) and bismuth germanium oxide (BGO; nucleon density 4.3 × 10^24^ cm^−3^) are used as the test material in recent works^19,22^. Because the possible signals are expected to be weak, a high-sensitivity magnetometer like a Spin-Exchange Relaxation Free (SERF) device^30–33^ is desired.

In this paper, we report a new measurement of *V*_4+5_ by using a K–Rb–^21^Ne comagnetometer^34^, and a tungsten test ring. Tungsten has a nucleon density of 1.15 × 10^25^ cm^−3^, which is among the highest-density practically-available non-magnetic materials. The sensitivity of SERF comagnetometers to pseudomagnetic fields acting on the sensor nuclei has been demonstrated to be better than 1 fT/Hz^1/2^^33,35^, which is one order of magnitude more sensitive than the “spin-based amplifier” demonstrated recently^19^. With these advantages, limits on the *V*_4+5_ neutron-nucleon and proton-nucleon interactions have been achieved.

## Results

### Experimental setup

**Fig. 1:**
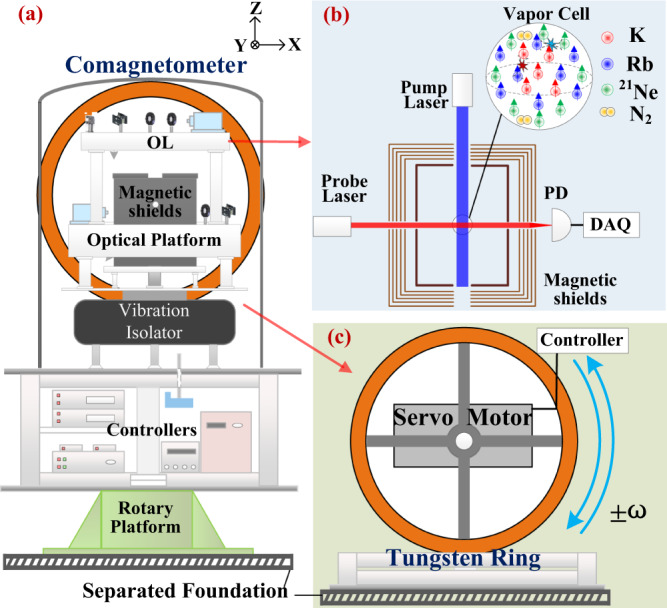
The experimental setup. **a** The structure of the whole equipment. The tungsten ring (nucleon source) is placed at the back of the comagnetometer along $\hat{y}$-axis. The center of the ring and the center of the comagnetometer cell are located at the same height. The comagnetometer is placed on a rotary platform that is isolated from the foundation of the tungsten ring. **b** The comagnetometer setup. The K, Rb, and ^21^Ne atoms are spin-polarized along $\hat{z}$-axis (vertical) by the pump laser light. The transverse magnetic field can be measured with the probe laser light. The fluctuations of light intensities, frequencies, cell temperature are reduced with feedback. OL, optical elements and lasers. PD, photodetector. DAQ, data acquisition. The external magnetic field and magnetic noise are suppressed by shielding the vapor cell in a five-layer *μ*-metal shield and a ferrite shield. **c** The tungsten ring. The ring, with its duralumin support, is driven with a servo motor. It can rotate clockwise and counterclockwise.

The experimental setup is shown in Fig. 1. A tungsten–aluminum (W–Al) ring, composed of tungsten wires and its ring-shape duralumin support, serves as the nucleon source and is located behind a K–Rb–^21^Ne comagnetometer. Driven with a servo motor, the W–Al ring can rotate clockwise or counterclockwise. The rotation axis of the ring is coaxial with the vapor cell along the $\hat{y}$ direction, along which the comagnetometer has the highest sensitivity. The angular position of the ring is monitored with a photoelectric encoder. If it exists, the pseudomagnetic field induced by the W–Al ring can be measured with the comagnetometer.

The K–Rb–^21^Ne comagnetometer is similar to that of Refs. [35,36]. Hybrid optical pumping is utilized to improve the polarization homogeneity of alkali spins and hyperpolarization efficiency of noble-gas spins, where the optically thin K atoms are optically polarized with a circularly polarized K D1-line laser along the $\hat{z}$-axis and are used to polarize the optically thick Rb atoms via spin-exchange (SE) collisions between K and Rb atoms. With the help of Rb and K, the ^21^Ne nucleus can be polarized through the spin-exchange-optical-pumping mechanism^37^. The precession of the Rb polarization is measured via optical rotation of a linearly polarized laser beam propagating along the $\hat{x}$-axis. The frequency of the laser is detuned from the Rb D1-line towards lower frequencies by about 240 GHz. Here the optically thick Rb ensemble is used for probing instead of the optically thin K ensemble, in order to improve the signal-to-noise ratio. The precession of the ^21^Ne nuclei is probed with Rb atoms, and detected via optical rotation of the probe laser beam. The K–Rb–^21^Ne comagnetometer is operated in the self-compensation regime to suppress magnetic noise. It is also operated in the SERF regime, which results in its high sensitivity to pseudomagnetic signals^32^.

### Principle

After zeroing the normal magnetic field, the leading terms in the comagnetometer signal in the self-compensation regime is given by^15^

4 $$S=K\frac{{\gamma }_{e}{P}_{z}^{e}}{{R}_{{{{{{{{\rm{tot}}}}}}}}}^{e}}\left({b}_{y}^{{{{{{{{\rm{Ne}}}}}}}}}-{b}_{y}^{e}+\frac{{{{\Omega }}}_{y}}{{\gamma }_{{{{{{{{\rm{Ne}}}}}}}}}}\right),$$

where *γ*_*e*_ and *γ*_Ne_ are the gyromagnetic ratios of electrons and ^21^Ne nuclei; ${P}_{z}^{e}$ and ${R}_{{{{{{{{\rm{tot}}}}}}}}}^{e}$ are the equilibrium spin polarization and transverse spin relaxation rate of alkali atoms, respectively; *K* is a factor to transform the ${P}_{x}^{e}$ to the output electric signal *S*; ${b}_{y}^{{{{{{{{\rm{Ne}}}}}}}}}$ and ${b}_{y}^{e}$ are the exotic fields along the $\hat{y}$ axis that couple to ^21^Ne and alkali atoms, Ω_*y*_ is the inertial rotation rate in $\hat{y}$ axis. Deploying in Eq.(4), we utilize the inertial rotation to calibrate the comagnetometer response to exotic fields, which is summarized by a s factor ${\kappa }_{n}\equiv K{\gamma }_{e}{P}_{z}^{e}/{R}_{{{{{{{{\rm{tot}}}}}}}}}^{e}$.

If we consider the specific coupling to fermions, the pseudomagnetic field on the ^21^Ne nuclei can be written as

5 $${b}_{y}^{{{{{{{{\rm{Ne}}}}}}}}}={B}_{p}^{n}{\zeta }_{n}^{{{{{{{{\rm{Ne}}}}}}}}}+{B}_{p}^{p}{\zeta }_{p}^{{{{{{{{\rm{Ne}}}}}}}}},$$

where ${\zeta }_{n}^{{{{{{{{\rm{Ne}}}}}}}}}=0.58$ and ${\zeta }_{p}^{{{{{{{{\rm{Ne}}}}}}}}}=0.04$ are the fraction factors for neutron and proton polarization in the ^21^Ne nucleus, respectively^15,38^, and ${B}_{p}^{p}$ and ${B}_{p}^{n}$ are the exotic fields acting on the proton and neutron, respectively. To detect ${b}_{y}^{{{{{{{{\rm{Ne}}}}}}}}}$, a quantum nondemolition approach is used. First, the precession of ^21^Ne nuclear spin under the ${b}_{y}^{{{{{{{{\rm{Ne}}}}}}}}}$ is transferred to the Rb atoms through the Fermi-contact interaction. By measuring the precession of Rb atoms based on optical rotation, one can measure the ${b}_{y}^{{{{{{{{\rm{Ne}}}}}}}}}$.

### Data taking and simulation

The signal from the comagnetometer, as well as the angular position of the ring, are recorded with a data-acquisition (DAQ) device. Data for an individual set are taken continuously for four hours, and 26 datasets are collected in total. Between the datasets the co-magnetometer performance is optimized. The parameters of the comagnetometer, including the cell temperature, laser power, laser frequency etc., are monitored and feedback controlled throughout the experiments to ensure its stability.

**Fig. 2:**
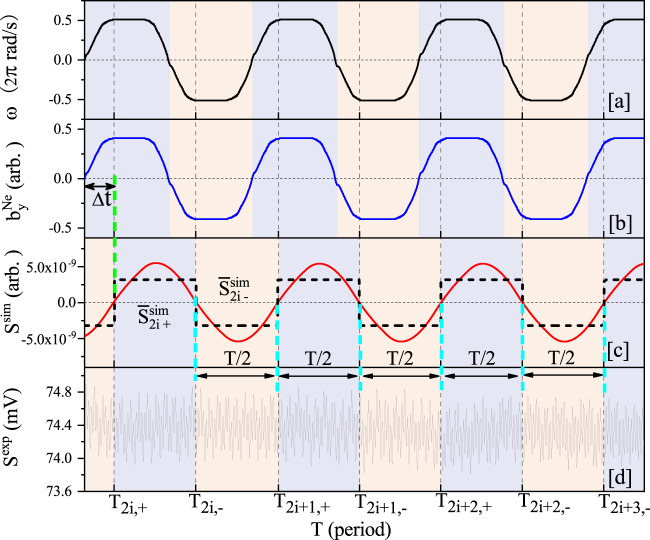
Data simulation and collection. **a** A representative measured angular velocity of the ring, *ω*(*t*). **b** The simulated pseudomagnetic field ${b}_{y}^{{{{{{{{\rm{Ne}}}}}}}}}$. **c** The simulated response of the comagnetometer ${S}^{{{{{{{{\rm{sim}}}}}}}}}$, which is delayed by Δ*t* with respect to the ${b}_{y}^{{{{{{{{\rm{Ne}}}}}}}}}$. The ${\bar{S}}_{2i,+}^{{{{{{{{\rm{sim}}}}}}}}}$ and ${\bar{S}}_{2i,-}^{{{{{{{{\rm{sim}}}}}}}}}$ are the average of each positive and negative half cycles, respectively. **d** The measured signal of the SERF comagnetometer.

To optimize the sensitivity to the hypothetical force, it is modulated by modulating the rotation frequency of the tungsten ring. This way, the exotic signal is shifted into a frequency region with optimum noise performance of the co-magnetometer. The angular velocity of the ring ${\omega }^{\exp }$ is measured with a high-precision photoelectric encoder and shown in Fig. 2a. The measured angular velocity is fitted with sinusoidal harmonics to obtain the amplitude (${\omega }_{\max }=3.774$ rad/s) and frequency (0.8369 Hz) of the fundamental harmonic.

The ${b}_{y}^{{{{{{{{\rm{Ne}}}}}}}}}$ induced by the test material can be simulated using Eq. (3) and Eq. (5). The major input parameters for the simulation are listed in Table 1, and the simulated ${b}_{y}^{{{{{{{{\rm{Ne}}}}}}}}}$ is shown in Fig. 2b. When calculating ${b}_{y}^{{{{{{{{\rm{Ne}}}}}}}}}$, a coupling constant ${f}_{4+5}^{(0)}$ is assumed. In Fig. 2b, it is set to be ${f}_{4+5}^{(0)}=1$.

**Table 1:** The experimental parameters and their error budget for the coupling coefficient

Parameter	Value	${{\Delta }}{f}_{4+5}^{\exp}$(×10^−21^)^a^
W–Al ring R (m)	0.475 ± 0.001	<0.001
Ring to cell center *X* (m)	0.478 ± 0.002	0.002
Ring to cell center *Y* (m)	0.000 ± 0.002	<0.001
Ring to cell center *Z* (m)	0.000 ± 0.002	<0.001
W–Al ring M (kg)	15.38 ± 0.05	0.001
${\omega }_{\max }$ (rad/s)	3.774 ± 0.001	<0.001
Calibrated *κ*_*n*_ (*μ*V/fT)	1.67 ± 0.05	0.016
Modulation freq. (Hz)	0.8369 ± 0.0001	
Phase shift (deg)	−67^∘^ ± 2^∘^	<0.001
Final ${f}_{4+5}^{\exp }$	−0.54	±0.02 (syst.)
(*λ* = 10 m)		±1.62 (stat.)^b^

^a^The contribution to the error budget of ${f}_{4+5}^{n}$ at *λ* = 10 m

^b^Error contribution from the uncertainties of the parameters listed above.

In Fig. 2c, the response of the comagnetometer ${S}^{{{{{{{{\rm{sim}}}}}}}}}$ is presented. This response signal is simulated based on Eq. (7) with parameters measured in the experiment. Because the main component of the ${b}_{y}^{{{{{{{{\rm{Ne}}}}}}}}}$ is at the fundamental harmonic and the bandwidth of the comagnetometer is narrow, the comagnetometer is mainly sensitive to the first harmonic component of ${b}_{y}^{{{{{{{{\rm{Ne}}}}}}}}}$, which results in the approximately sinusoidal shape of ${S}^{{{{{{{{\rm{sim}}}}}}}}}$. Compared with ${b}_{y}^{{{{{{{{\rm{Ne}}}}}}}}}$, the ${S}^{{{{{{{{\rm{sim}}}}}}}}}$ has a phase shift Δ*ϕ* = −67^∘^ ± 2^∘^ due to the phase response of the comagnetometer (corresponding to a time delay of Δ*t* = 0.222 ± 0.007 s). In Fig. 2d, the corresponding experimental data ${S}^{\exp }$ are shown. There are slight beating patterns in the experimental data, which are due to the resonant vibrations of the isolation station and the table supporting the comagnetometer.

### Limits on exotic forces

The coupling constant can be found by

6 $${f}_{4+5}={f}_{4+5}^{(0)}\frac{{\bar{b}}^{\exp }}{{\bar{b}}^{{{{{{{{\rm{sim}}}}}}}}}},$$

where the ${\bar{b}}^{\exp }$ and ${\bar{b}}^{{{{{{{{\rm{sim}}}}}}}}}$ are exotic fields from the experiment and simulation respectively. The details of the data analysis can be found in the Supplementary Note 3.

**Fig. 3:**
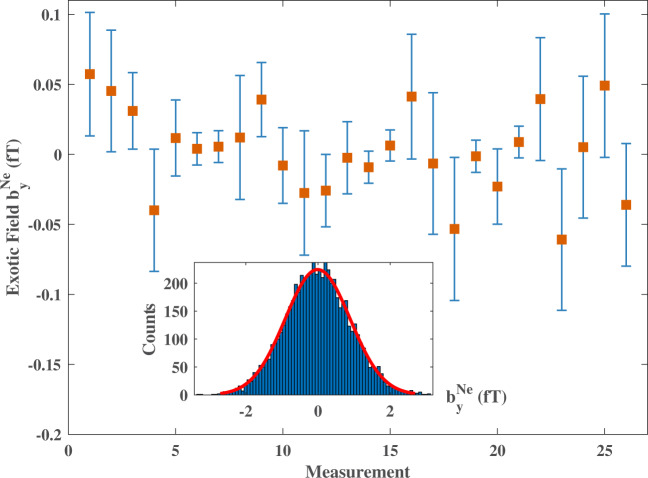
Experimental results of the ${b}_{y}^{{{{{{{{\rm{Ne}}}}}}}}}$. Each point represents an average of a 4-h dataset, ${\bar{b}}_{y}^{{{{{{{{\rm{Ne}}}}}}}}}$. The error bars represent the statistical and systematic error of the comagnetometer combined in quadrature. Histogram of ${\bar{b}}_{y}^{{{{{{{{\rm{Ne}}}}}}}}}$ is shown in the inset, with the red curve being a Gaussian fit, and ${\bar{\chi }}^{2}=1.14$. The exotic field ${b}_{y}^{{{{{{{{\rm{Ne}}}}}}}}}$ is measured to be (2.4 ± 7.1)aT.

**Fig. 4:**
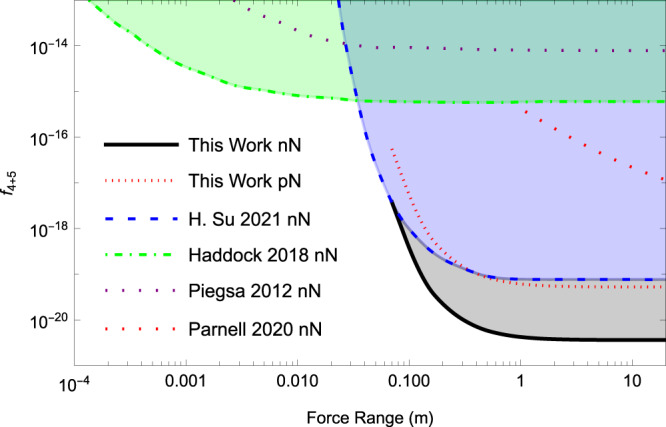
The experimental limits on *f*_4+5_. The “n'', “p'', and “N” represent the neutron, proton, and average nucleon contribution respectively. The blue dashed line, “H.Su 2021'', is from Ref. [19], the green dashed-dotted line, “Haddock 2018'', is from Ref. [24], the yellow dotted line, “Piegsa 2012", is from Ref. [25], the red dashed line, “Parnell 2020", is from Ref. [50]. The black solid line and red dotted line represent our new results for “nN" and “pN" respectively.

A typical ${\bar{S}}^{\exp }$ distribution, deduced from 4-hour data, is shown in the inset of Fig. 3. Note that the proton fraction of polarization in the Rb atom is ${\zeta }_{p}^{Rb}=0.31$^39^, but the Rb atoms’ energy sensitivity is three orders of magnitude smaller than that of ^21^Ne^15,40^ (see the Supplementary Note 2B). Thus we only consider the proton spin in ^21^Ne and ignore that in Rb atoms. Using all the 104-hour data, the pseudomagnetic field ${b}_{y}^{{{{{{{{\rm{Ne}}}}}}}}}$ is measured to be (2.4 ± 7.1)aT. This results in new limits on the dimensionless coupling factor of the new force ∣*f*_4+5_∣ ≤ 3.4 × 10^−21^ with 95% confidence level at the force range of 10 m. The limits on the coupling between the source nucleon with the ^21^Ne neutron and proton are set to be $|{f}_{4+5}^{n} |=|{f}_{4+5}|/{\zeta }_{n}^{{{{{{{{\rm{Ne}}}}}}}}}\,\le \,5.9\times 1{0}^{-21}$ and $|{f}_{4+5}^{p} |=|{f}_{4+5}|/{\zeta }_{p}^{{{{{{{{\rm{Ne}}}}}}}}}\,\le \,8.5\times 1{0}^{-20}$ respectively. Our experiment results and a comparison with the results in the literature are shown in Fig. 4. Our result on the coupling with neutron is more than an order of magnitude better than that of previous works. The limits on exotic coupling with proton is set by this work for the first time to the best of our knowledge. For the coupling to electron spins, our experimental result is comparable to that of a recent work in Ref. [29].

It may be useful to consider specific cases for coupling, using the fact that ${f}_{4+5}=\frac{1}{2}{g}_{A}{g}_{A}-\frac{3}{2}{g}_{V}{g}_{V}$. Assuming *g*_*A*_*g*_*A*_ = 0, we have $|({\zeta }_{p}^{{{{{{{{\rm{Ne}}}}}}}}}{g}_{V}^{p}+{\zeta }_{n}^{{{{{{{{\rm{Ne}}}}}}}}}{g}_{V}^{n})({\zeta }_{p}^{W}{g}_{V}^{p}+{\zeta }_{n}^{W}{g}_{V}^{n})|\,\le \,2.3\times 1{0}^{-21}$, where ${\zeta }_{n}^{W}=0.588$ and ${\zeta }_{p}^{W}=0.412$ are the neutron and proton mass contribution in the W–Al mass source. If we assume ${g}_{V}^{p}=0$, we get a limit on $|{g}_{V}^{n}|\,\le \,8.2\times 1{0}^{-11}$. Conversely, if we assume ${g}_{V}^{n}=0$, we get $|{g}_{V}^{p}|\,\le \,3.7\times 1{0}^{-10}$. The coupling limit $|{g}_{A}^{p}|\,\le \,6.4\times 1{0}^{-10}$ and $|{g}_{A}^{n}|\,\le \,1.4\times 1{0}^{-10}$ is also set using the same method.

**Fig. 5:**
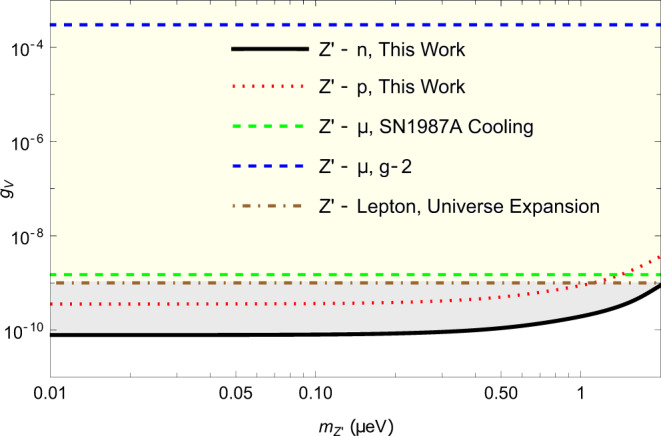
The experimental limits on the vector coupling constant *g*_*V*_ of the Z’ particle. The black solid line and red dotted line are set by this work by searching for the exotic spin-dependent force. The blue dashed line is set by the *g*−2 experiment^41,44,45^, the brown dotted-dashed line is set by the neutrino production in the expansion of the early universe^41^, the green dashed line, is from the analysis of the supernova SN1987 cooling^44^. The shaded areas are the parameter space excluded by the corresponding experiments.

A comparison of the limits on *g*_*V*_, the vector coupling constant between Z’ and standard model particles, between this SVD force result and other results including the cosmology and astronomy is shown in Fig. 5. The ‘Z’-Lepton Universe Expansion’ line is excluded by the effective number of neutrino species Δ*N*_nef_ ≈ 0.2 in the early universe^41^. The authors of this work assume that Z’ can decay to neutrinos and affect the expansion of the universe. This model can relax the 3*σ* tension of Hubble constants, i.e., the discrepancy between local measurements and temperature anisotropies of the cosmic microwave background^41–43^. The ‘Z’-*μ*, *g* − 2’ is excluded by the muon *g*−2 experiment^41,44,45^. Note that the anomalous magnetic moment of muon can also be used to search for the spin-0 boson, such as axion like particles^6^. The ‘Z’-*μ*, SN1987A Cooling’ is excluded by the supernova SN1987A, assuming the new gauge boson Z’ decreases the cooling time^44^. Our results represent more than one order of magnitude tighter constraints than previous ones.

## Discussion

The main advantage of this experiment compared to that in Ref. [19] are attributed to the high nucleon density of the nucleon source and the ultrahigh sensitivity of the comagnetometer. Tungsten has nucleon density approximately four times that of BGO, and our comagnetometer sensitivity is approximately one order of magnitude better than the ^129^Xe based magnetometer used in Ref. [19].

The comagnetometer can also be used to search for many terms of spin-spin-velocity-dependent forces if one can use a spin-polarized source, such as a pure-iron shielded SmCo_5_ electron-spin source^14^. For four terms of the spin-spin-velocity-dependent forces, new limits on electron-neutron and electron-proton interaction can be set in the force range ≥1 cm^46^, which would complement the results of^17^ for interaction ranges exceeding 1 km.

The techniques used in this work can be used to search for a broad class of beyond-standard-model particles^10,11,28^. The search for such particles is motivated, among other things, by the attempt to understand the composition of the dark sector (dark matter and dark energy). However, similar to other spin-dependent force searches, it does not, in any way, rely on specific local dark-sector properties, e.g., the local dark-matter density. If the exotic-force mediator is a Z’-boson, the interactions could violate parity, however, the current experiment does not exploit parity-violating effects. Indeed, the interaction in Eq. (2) is P- and T-even.

In conclusion, in this work, we have searched for exotic spin- and velocity-dependent interactions and, for the force range larger than several centimeters, improved the limits on the mass interactions with neutrons by more than an order of magnitude, while also setting a stringent limit on mass interactions with protons. This result demonstrates that the spin-dependent force approach is competitive in terms of sensitivity to new bosons with the cosmological and astronomical searches. Z’ can also be searched through pseudovector parity-violation couplings ^47,48^.

## Methods

### Calibration

**Fig. 6:**
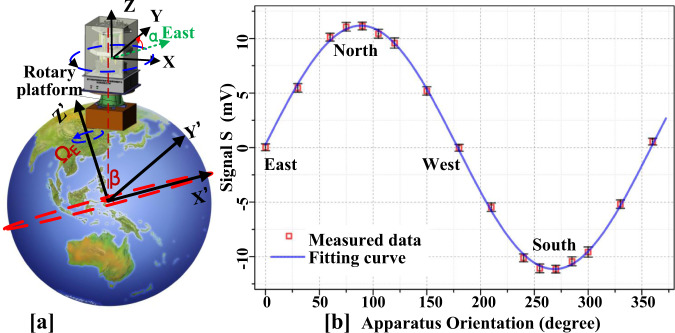
Calibration of the comagnetometer. **a** The comagnetometer is rotated by a rotary platform. The sensitive axis ($\hat{y}$) of comagnetometer is directed along different direction. Hence, the projection of Earth rotation along $\hat{y}$ can be used to calibrate the comagnetometer. *β* is the latitude of the lab, *α* is the orientation angle of $\hat{y}$ with respect to the east direction in the horizontal plane. **b** The measured dots at different direction are fitted by a sinusoidal function. The error bars represent statistical errors (standard deviations).

The Earth rotation speed Ω_*E*_ = 7.292 × 10^−5^ rad/s is used to calibrate the system. Mounted on a precision rotary platform, the apparatus can rotate in the horizontal plane as shown in Fig. 6 [a]. The Ω_*y*_ in Eq. (4) can be written as ${{{\Omega }}}_{y}={{{\Omega }}}_{E}^{h}\sin (\alpha )$, where ${{{\Omega }}}_{E}^{h}={{{\Omega }}}_{E}\cos (\beta )$ is the projection of **Ω**_*E*_ in the horizontal plane, *β* = 39.983^∘^ is the latitude of the laboratory, and *α* is the relative azimuth angle of the sensitive $\hat{y}$ axis of the comagnetometer. Therefore, by fitting the measured signals with $S(\alpha )={\kappa }_{n}{{{\Omega }}}_{E}^{h}\sin (\alpha )/{\gamma }_{n}$, the calibration factor *κ*_*n*_ can be obtained. As shown in Fig. 6 [b], the calibration factor at this near-DC frequency is measured to be *κ*_*n*_(DC)=(4.18 ± 0.07) × 10^−6^ V/fT with the ${\bar{\chi }}^{2}=0.687$.

In the self-compensation comagnetometer, the frequency responses to normal magnetic fields, inertial rotations (*B*_*x*/*y*_, and Ω_*x*/*y*_) and exotic fields (${b}_{y}^{{{{{{{{\rm{Ne}}}}}}}}}$ and ${b}_{y}^{e}$) are determined by four parameters, which are the Fermi-contact-interaction fields between noble-gas atoms and alkali atoms ($\lambda {M}_{0}^{e}{P}_{z}^{e}$ and $\lambda {M}_{0}^{n}{P}_{z}^{n}$)^49^, and the transverse relaxation rates (1/*T*_2*e*_ and 1/*T*_2*n*_). These four parameters are independently measured to be (110.6 ± 2.2) nT, (579.4 ± 2.1) nT, (3.7 ± 0.4) × 10^3^ /s, and (0.063 ± 0.007) /s, respectively^36^. Particularly, the noble-gas nuclear spins and alkali electron spins are strongly coupled around the self-compensation point, such that the damping rate of nuclear spins is sped up by electron spins, while electron spins are slowed down^36^. The damping rate of nuclear spins is measured to be 2*π* × (0.907 ± 0.006)/s by fitting the damping oscillation response to step magnetic field excitation, which is significantly larger than the 1/*T*_2*n*_ owing to the coupling between nuclear spins and electron spins. More details about the analysis of the comagnetometer can be found in Supplementary Note 2 and 3.

### Simulation

The spin dynamics of the comagnetometer could be described by the coupled Bloch equations^32,36^:

7 $$\begin{array}{ll}\frac{\partial {{{{{{{{\bf{P}}}}}}}}}^{e}}{\partial t}=&\frac{{\gamma }_{e}}{Q}\left({{{{{{{\bf{B}}}}}}}}+\lambda {M}_{0}^{n}{{{{{{{{\bf{P}}}}}}}}}^{n}+{{{{{{{{\bf{b}}}}}}}}}^{e}+{{{{{{{\boldsymbol{\Omega }}}}}}}}\frac{Q}{{\gamma }_{e}}\right)\times {{{{{{{{\bf{P}}}}}}}}}^{e}\\ &+\frac{{P}_{z0}^{e}\hat{z}-{{{{{{{{\bf{P}}}}}}}}}^{e}}{Q\{{T}_{1e},{T}_{2e},{T}_{2e}\}},\\ \frac{\partial {{{{{{{\bf{{P}}}}}}}^{n}}}}{\partial t}=&{\gamma }_{{{{{{{{\rm{Ne}}}}}}}}}\left({{{{{{{\bf{B}}}}}}}}+\lambda {M}_{0}^{e}{{{{{{{{\bf{P}}}}}}}}}^{e}+{{{{{{{{\bf{b}}}}}}}}}^{{{{{{{{\rm{Ne}}}}}}}}}+\frac{{{{{{{{\boldsymbol{\Omega }}}}}}}}}{{\gamma }_{{{{{{{{\rm{Ne}}}}}}}}}}\right)\times {{{{{{{{\bf{P}}}}}}}}}^{{{{{{{{\bf{n}}}}}}}}}\\ &+\frac{{P}_{z0}^{n}\hat{z}-{{{{{{{{\bf{P}}}}}}}}}^{n}}{\{{T}_{1n},{T}_{2n},{T}_{2n}\}},\end{array}$$

where *Q* is the slowing-down factor arising from spin-exchange collisions and hyperfine interaction, **P**^**e**^ and **P**^**n**^ are the spin polarizations of alkali electron and ^21^Ne nuclear, respectively. **B** and **Ω** are external magnetic field and inertial rotation. The Fermi-contact interaction between alkali atoms and ^21^Ne atoms can be described by an effective magnetic field $\lambda {M}_{0}^{{{{{{{{\rm{e}}}}}}}},{{{{{{{\rm{n}}}}}}}}}{{{{{{{{\bf{P}}}}}}}}}^{{{{{{{{\rm{e}}}}}}}},{{{{{{{\rm{n}}}}}}}}}$, where ${M}_{0}^{n}$ (${M}_{0}^{e}$) is the maximum magnetization of ^21^Ne nucleon (alkali electron)^49^. For a uniformly spin-polarized spherical cell, *λ* = 8*πκ*_0_/3, where *κ*_0_ is the enhancement factor. *T*_1*e*_ and *T*_2*e*_ are the longitudinal and transverse relaxation rates for alkali electron spin, respectively, and *T*_1*n*_ and *T*_2*n*_ are the longitudinal and transverse relaxation times for the ^21^Ne nucleon spin.

**Fig. 7:**
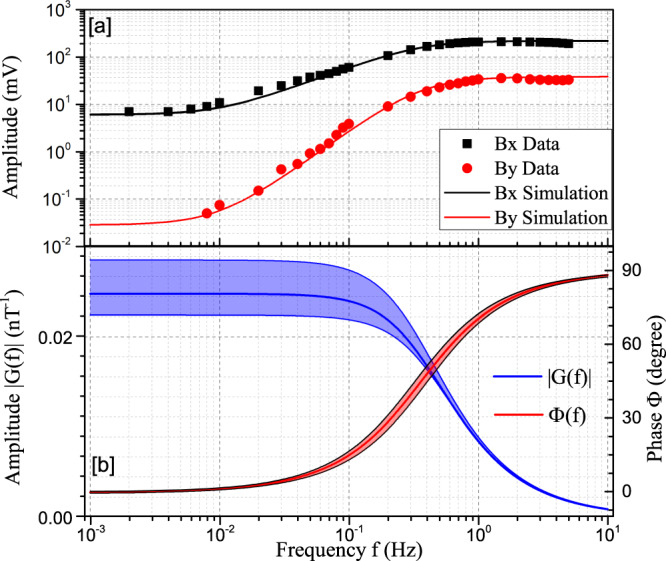
Frequency responses of the comagnetometer. **a** A comparison of the measured frequency responses to normal magnetic field and the simulated results. The simulated results are calculated with Eq.(7) based on measured parameters ($\lambda {M}_{0}^{e}{{{{{{{{\bf{P}}}}}}}}}_{z}^{e}$, $\lambda {M}_{0}^{n}{{{{{{{{\bf{P}}}}}}}}}_{z}^{n}$, *T*_2*e*_ and *T*_2*n*_) with only one free parameter to describe the scale. The measured data agree well with the simulation of the Bloch equation Eq. (7), which confirms the validity of the simulation. **b** The simulated amplitude-frequency response (blue) and phase-frequency response (red) to exotic field b${}_{y}^{{{{{{{{\rm{Ne}}}}}}}}}$. The uncertainty bands of the phase and amplitude are calculated based on the uncertainties of the measured parameter.

As shown in Fig. 7 [a], to further verify the validity of the parameters $\lambda {M}_{0}^{e}{P}_{z}^{e}$, $\lambda {M}_{0}^{n}{P}_{z}^{n}$, *T*_2*e*_, and *T*_2*n*_) and the frequency response model, the frequency responses to *B*_*y*_ and *B*_*x*_ are measured and compared with the simulated results with Eq. (7) based on these four parameters with only one free parameter to describe the scale. The measured signals are consistent with the simulated results. Therefore, the response of the comagnetometer ${S}^{{{{{{{{\rm{sim}}}}}}}}}$ to the exotic field ${b}_{y}^{{{{{{{{\rm{Ne}}}}}}}}}$ can be simulated by solving the Bloch Eqs. (7)^40^ with these verified parameters. The simulation result of the amplitude and phase response to ${b}_{y}^{{{{{{{{\rm{Ne}}}}}}}}}$ is shown in Fig. 7 [b]. The results are further used to correct the calibration factor *κ*_*n*_ and the phase retardation Δ*ϕ*. For the modulation frequency at 0.83681(1) Hz, the amplitude response is 0.40 ± 0.01 of that in DC. Meanwhile, the phase shift Δ*ϕ* is − 67^∘^ ± 2^∘^. The corrected calibration factor is *κ*_*n*_(0.84 Hz) = (1.67 ± 0.05) × 10^−6^ V/fT. A detailed analysis of the uncertainty of calibration factor can be found in Supplementary Note 3.

## Supplementary information

Supplementary Information

## Data Availability

The datasets generated during the current study are available from the corresponding author on request.
