# Supplementary material for: Constraints on exotic spin-velocity-dependent interactions
Source: Nat Commun. 2022 Nov 30;13:7387. doi: 10.1038/s41467-022-34924-z (PMC9712588; doi:10.1038/s41467-022-34924-z)
Supplement: Supplementary file 1 — Supplementary Information [file 41467_2022_34924_MOESM1_ESM.pdf]

Supplementary Information for:
"Constraints on Exotic Spin-Velocity-Dependent Interactions"

Kai Wei,[1,2,3] Wei Ji,[4,5,*] Changbo Fu,[6,†] Arne Wickenbrock,[4,5]
Victor V. Flambaum,[5,7] Jiancheng Fang,[1,2] and Dmitry Budker[4,5,8]

[1]*School of Instrumentation Science and Opto-electronics Engineering, Beihang University, Beijing, 100191, China*
[2]*Hangzhou Extremely Weak Magnetic Field Major Science and Technology Infrastructure Research Institute, Hangzhou, 310051, China*
[3]*Hangzhou Innovation Institute, Beihang University, Hangzhou, 310051, China*
[4]*Helmholtz-Institut, GSI Helmholtzzentrum fur Schwerionenforschung, Mainz 55128, Germany*
[5]*Johannes Gutenberg University, Mainz 55128, Germany*
[6]*Key Lab of Nucl. Phys. & Ion-beam Appl. (MoE), Inst. of Modern Phys., Fudan Univ., Shanghai 200433, China*
[7]*School of Physics, University of New South Wales, Sydney, New South Wales 2052, Australia*
[8]*Department of Physics, University of California, Berkeley, CA 94720-7300, USA*
∗*Corresponding Author: wei.ji.physics@gmail.com*
†*Corresponding Author: cbfu@fudan.edu.cn*

(Dated: November 7, 2022)

Supplementary Note 1. EXPERIMENT

A. Experimental Setup

The experimental setup is shown in the main text Fig.1. The tungsten-duralumin (W-Al) ring is composed of (11.20 ± 0.05) kg of tungsten wires wrapped on a ring-shape duralumin support, which has a total mass of (4.18 ± 0.01) kg. The tungsten has nucleon density of $1.15 \times 10^{25}\,\mathrm{cm}^{-3}$, and the duralumin has a nucleon density of $1.67 \times 10^{24}\,\mathrm{cm}^{-3}$. The inner radius of the tungsten ring is $R = (0.475 \pm 0.001)$ m. The height of the center of the ring is the same as the height of the center of the vapor cell, and the distance between the center of the ring and the center of the comagnetometer cell is $D_c = (0.478 \pm 0.003)$ m. Four duralumin supporting rods are installed to connect the aluminium-alloy spindle and the ring-shape support. The test material, including the tungsten ring and its duralumin holder, can all generate the exotic force. Based on the simulation, the ring part generates 90% of the exotic force, while the other moving parts in the device generate the rest. The W-Al ring is rotated by a precision servo motor. The rotation of W-Al ring is monitored with a photoelectric encoder.

The K-Rb-^{21}Ne comagnetometer (the Supplementary Fig. 1) is used to detect the effective magnetic field caused by the exotic force. A spherical glass cell is filled with 2500 torr of ^{21}Ne (70% isotope enriched) and 54 torr of N_2, as well as a small droplet of K and Rb atoms. The cell is heated to 200°C with an AC electric heater to reduce low-frequency magnetic interference. At 200°C, the number densities of K and Rb atoms are around $8\times10^{12}\,\mathrm{cm}^{-3}$ and $8\times10^{14}\,\mathrm{cm}^{-3}$ respectively, leading to a density ratio of about 1/100. The cell and the heater are enclosed inside a water-cooled vacuum chamber to improve the temperature stability and reduce air convection. The cell is placed inside a five-layer μ-metal magnetic shield to reduce the ambient magnetic field. Additionally, a Mn-Zn ferrite shield, which has high resistivity [1], is placed in the innermost layer to minimize the magnetic noise from the shielding material. After degaussing with a 60 A induction degaussing device, the residual magnetic fields and field gradient at the center of the magnetic shields are measured with a fluxgate magnetometer to be smaller than 1 nT and 1 nT/cm, respectively. These residual fields are further reduced using compensation coils.

Hybrid spin-exchange optical pumping is applied to polarize the alkali electron spin and noble gas nuclear spins. The K electron spins are polarized by the circularly-polarized K D1 pumping light. And then the optical-thick Rb atoms are polarized by spin-exchange collisions with the optically thick K atoms. The ^{21}Ne nuclear spins are further polarized by spin-exchange collisions with Rb atoms. Since we use hybrid optical pumping to improve the polarization homogeneity, higher pump laser intensity is required. Pump laser light from a tapered amplifier (about 3 W) is coupled into an optical fiber, resulting in 1.3 W in the clean spacial mode. The laser beam is intensity stabilized with a liquid crystal modulator and is expanded to cover the 12 mm diameter cell. The intensity of the incident pump light is about 564 mW/cm^2. Initially, the K-Rb-^{21}Ne atoms are pumped for at least five hours to reach the quasi-steady state with a leading field along $\hat{z}$ of about 900 nT before we start to execute the field-zeroing procedure. The comagnetometer works continuously for several weeks to ensure the stablility of the system.

Optical rotation, a quantum nondemolition measurement method, is utilized to measure the spin precession of Rb atoms. Linearly polarized laser light detuned towards lower frequencies by about 240 GHz from the D1 line of Rb is used to probe the spin precession of Rb atoms, originating from the precession of ^{21}Ne nucleon magnetization caused by the exotic field. Furthermore, to avoid low-frequency noise, the probe laser is modulated with a photoelastic modulator (PEM) and the signal is demodulated by a lock-in amplifier. The intensity of probe laser is actively stabilized with liquid crystal variable retarders using PID feedback control to suppress intensity-related noise. Meanwhile, to reduce the light-frequency noise, the probe laser is stabilized with a two-stage temperature controller. The measured background noise from the probe light is smaller than 1 fT/Hz$^{1/2}$ around 1 Hz. The photon shot noise is calculated to be about 0.2 fT/Hz$^{1/2}$. This means the probe noise is limited by

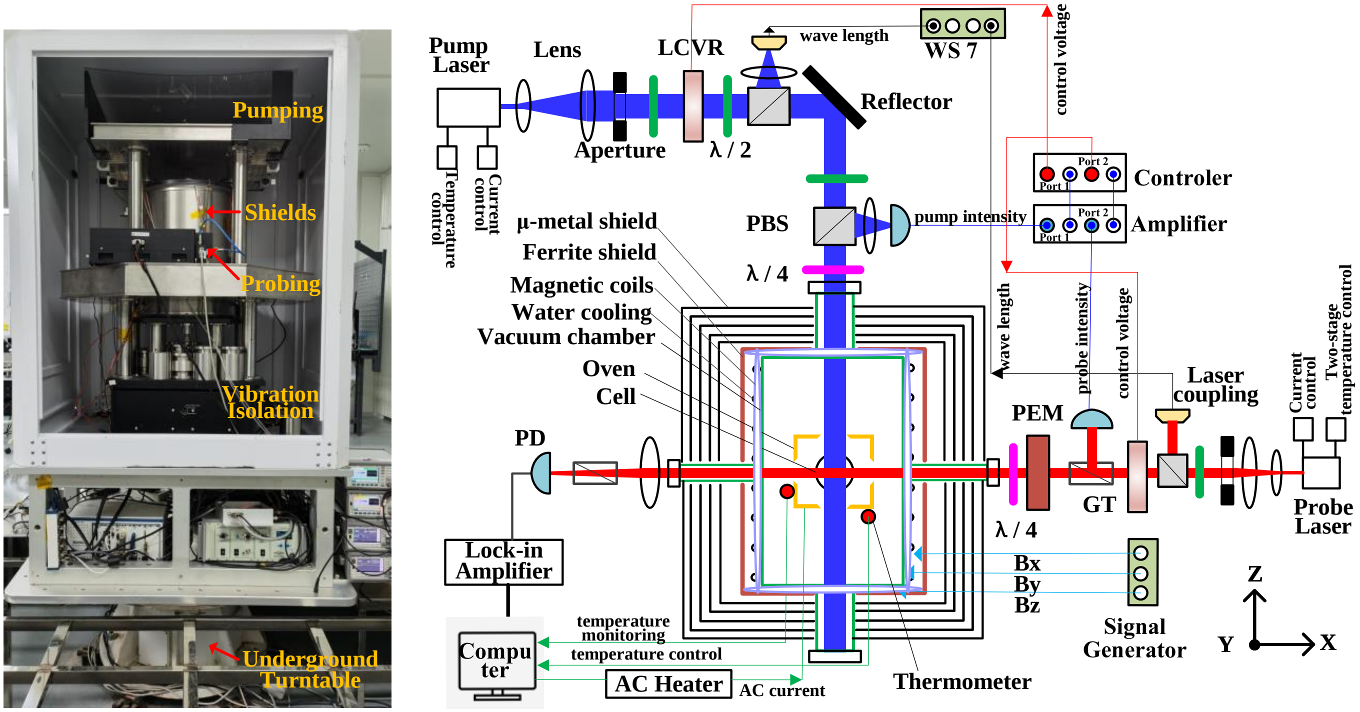

Supplementary Figure 1. The comagnetometer setup. The upper optical table is for optical pumping, while the lower one is for optical probing. The glass cell containing a small droplet of K-Rb, ^{21}Ne and N_2 is enclosed in an oven. The oven is installed in a vacuum chamber, which is also the frame for triaxial magnetic coils and water cooling tube. The pump and probe laser intensities are feedback controlled. LCVR: liquid crystal phase retarder; $\lambda/2$: half-wave plate; $\lambda/4$, quarter-wave plate; PBS: polarization beam splitter; PD, photoelectric detector; GT, Glen-Taylor prism; PEM, photoelastic modulator.

technical noises, such as noises related to unwanted birefringence and dichroism. To reduce the background noise, the optical polarization of probe laser is adjusted with a quarter-wave plate to compensate the birefringence of vacuum windows and the cell based on the response to magnetic field modulation.

Special efforts are made to reduce the air convection and drifts on the optical path. Firstly, we try to shorten the pumping and probing optical paths by using home-made small-size optical holders. Secondly, we try to avoid using reflectors to reduce the drift of light position due to reflection. Especially, we set the optical components of probe laser in a straight line to improve the stability. Finally, we enclose the pump and probe optics in acrylic boxes and send the laser beam into the vacuum chamber through vacuum tubes.

The comagnetometer is mounted on an underground high-precision turntable. The comagnetometer can be rotated by the turntable, such that the sensitive axis of the comagnetometer ($\hat{y}$) can be oriented to different direction in the horizontal plane. Therefore, the projection of Earth rotation on the horizontal plane can be used as to calibrate the comagnetometer. Experiment devices are installed on a stable rack. The customized nonmagnetic optical tables and the magnetic shields are mounted on vibration isolation.

B. Feedback control of parameters

The temperature of the vapor cell could affect the experiment in various ways. Hence, temperature stabilization is important for the experiment. We use a high-precision platinum resistor and a source measure unit (PXIe 4145, National Instruments) to measure the oven temperature. A LabView program is applied to acquire and analyse the measured value. Based on PID (proportional integral derivative) control, a control signal is sent to a home-made AC current generator to supply a controlled AC current (200 kHz) to the heating coils, which are printed in a flexible heat-resisting films and pasted on the oven with high thermal conductivity adhesive. The experiment temperature is set to 200°C. The short-term fluctuation is smaller than 0.005°C, while the long-term fluctuation is about 0.01°C for four hours. Furthermore, besides this platinum resistor for feedback control, another platinum resistor is pasted on the stem of the cell for monitoring the temperature. The monitoring resistor presents a slightly lower temperature of about 198.6°C and also with a high stability.

The pumping laser intensity is another important experimental parameter, which determines the spin polarizations of the alkali electrons and the noble-gas nuclei. We also employ PID control to suppress the fluctuation of pumping laser light intensity. As shown in the Supplementary Fig. 1, a liquid crystal phase retarder (LCVR) is utilized. The phase difference between the fast and slow axes can be changed by applying different voltage. The polarization of the input light is directed 45° with respect the fast axis of LCVR. After passing through the LCVR and a half-wave plate, the polarization of the light is changed according to the applied voltage of LCVR. And a PBS is used as an analyzer to determine the intensity of passing light. The other beam light of the analyzer PBS is measured with photoelectric detector to feedback the actual light intensity. The fluctuation of pumping laser intensity is about 0.1%. The probe laser intensity, whose fluctuation would cause the fluctuation of the scale factor κ_n, is also stabilized by using an LCVR.

The pumping laser frequency would impact the spin polarizations as well, and also change the light shift experienced by alkali atoms. We use saturated absorption in a separate cell to stabilize the frequency of pumping laser, locking the frequency of the laser to one hyperfine component of the K D1 line. Although tuning the laser to the resonance frequency of K D1 line results in a slight light shift of Rb transitions, such a small constant light shift only causes slight crosstalk between input signals in $\hat{x}$ axis (b_x^{Ne}) and $\hat{y}$ axis (b_y^{Ne}). This crosstalk is very small (only 10% of b_y^{Ne}). Together with the fact that the exotic field along the $\hat{x}$-axis, arising because of the imperfection misalignment, is less than 4×10^{-3} of that along $\hat{y}$ axis, this light-shift crosstalk is about 4×10^{-4}, which is negligible. The fluctuation of pumping laser frequency $\delta\nu_{\text{pu}}$ is about 5 MHz for four hours after stabilization.

The probe laser frequency would affect the scale factor κ_n, whose fluctuation should be suppressed. Because we tune the probe laser frequency far away from the resonance frequency by about 240 GHz to optimize the response, the saturated absorption frequency stabilization cannot be applied here. We improve the stability of the laser itself. Typically, a thermoelectric cooler (TEC) is used to stabilize the temperature of the laser chip, hence stabilize the frequency of the laser. We add another TEC and temperature control to further improve the stability of light frequency. We use a wavelength meter to monitor the light frequency. The probe laser frequency is stable at the level better than 50 MHz for four hours.

C. Modulation of the exotic force

To reduce the effects of low-frequency drifts of the comagnetometer and the mass source, the rotation of W-Al ring is modulated at about 0.84 Hz. The modulated rotation pattern is shown in Fig. 2 in the main manuscript. The angular velocity $\omega(t)$ of the W-Al ring is designed to have the following pattern: acceleration time interval $[\omega(t)=\alpha t,\ (n-\frac{2}{14})T\le t\le(n+\frac{2}{14})T]$; positive time interval (Clockwise) $[\omega(t)=\omega_{\text{max}},\ (n+\frac{2}{14})T\le t\le(n+\frac{5}{14})T]$; deceleration time interval $[\omega(t)=\omega_{\text{max}}-\alpha t,\ (n+\frac{5}{14})T\le t\le(n+\frac{9}{14})T]$; negative time interval (Counter Clockwise) $[\omega(t)=-\omega_{\text{max}},\ (n+\frac{9}{14})T\le t\le(n+\frac{12}{14})T]$, where α is the acceleration rate and T is the period for each modulation cycle. Due to the large moment of inertia of the ring, the maximum acceleration rate of the servo motor is limited to $\alpha=6\pi$ rad/s^2.

The modulated rotation rate of the W-Al ring is measured with a precision photoelectric encoder. The measured rotation speed is shown in the Supplementary Fig. 2 (a), and is fitted with sinusoidal harmonics to get the amplitude and frequency of the fundamental harmonic, which are 0.6006(2) rot/s and 0.8369(1) Hz respectively. The amplitude of the fundamental harmonic (0.84 Hz) is about 7 times larger than that of the 2.51 Hz. Besides, the ^{21}Ne atoms in the comagnetometer have a relatively narrow bandwidth, thus the comagnetometer is insensitive to the higher frequencies b_y^{Ne}. Therefore, although the rotation rate features a trapezoidal wave pattern, the effective exotic field sensed by the comagnetometer is the 0.84 Hz sinusoidal signal. The contribution of 2.51 Hz signal is smaller than that of the fundamental harmonic (0.84 Hz) signal by a factor of more than 20. We also compare the result of the fit and that of the FFT analysis. The FFT spectrum is shown in the Supplementary Fig. 2 (b). The frequencies of the fundamental harmonic signal are the same with these two methods. The amplitude of the fundamental harmonic signal of the FFT is 1.5% smaller than the fit result, which is probably due to the frequency leakage.

Supplementary Note 2. ANALYSIS OF THE SYSTEM

A. Analysis of SERF Comagnetometer

This section presents a detailed analysis of the SERF Comagnetometer. The comagnetometer uses ^{21}Ne noble gas and Rb-K alkali atoms, which are strongly coupled to operate in the spin-exchange relaxation free (SERF) regime as well as self-compensation (SC) regime simultaneously. The low-density K atoms act as a mediator to transfer the spin momentum of pumping photon to the high-density Rb atoms, avoiding strong absorption of the resonant pumping laser by high-density alkali atoms. The interactions between alkali atoms and ^{21}Ne atoms are dominated by the high-density Rb atoms. The coupled spin

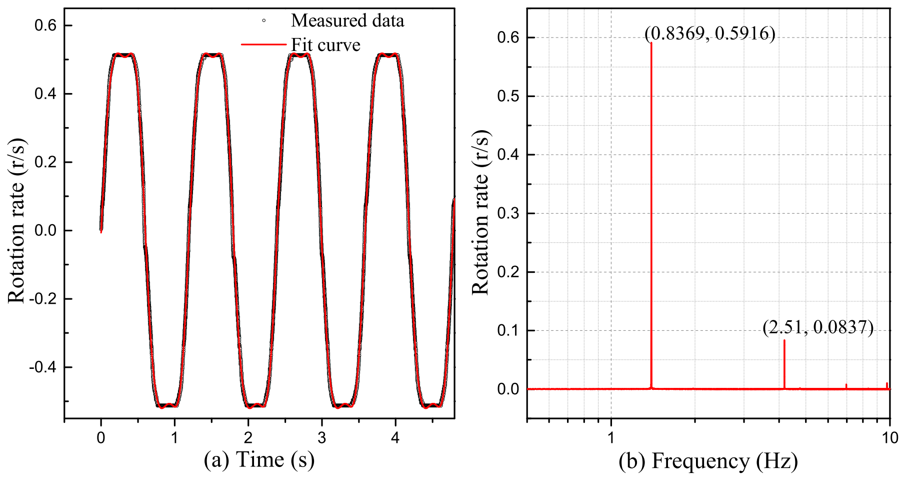

Supplementary Figure 2. The rotation speed of the spin source. (a) The measured rotation speed is fitted by sinusoidal harmonics. (b) The FFT result of the measured rotation speed.

dynamics of Rb-^{21}Ne in a uniformly magnetized spherical cell are described by the coupled Bloch equations,

$$\begin{aligned}\frac{\partial \mathbf{P^e}}{\partial t} &= \frac{\gamma_e}{Q}\left(\mathbf{B}+\lambda M_0^n \mathbf{P}^n+\mathbf{b^e}+\mathbf{\Omega}\frac{Q}{\gamma_e}\right)\times \mathbf{P^e}+\frac{P_{z0}^e\hat{z}-\mathbf{P^e}}{Q\{T_{1e},T_{2e},T_{2e}\}},\\ \frac{\partial \mathbf{P^n}}{\partial t} &= \gamma_{\rm Ne}\left(\mathbf{B}+\lambda M_0^e \mathbf{P^e}+\mathbf{b}^{\rm Ne}+\frac{\mathbf{\Omega}}{\gamma_{\rm Ne}}\right)\times \mathbf{P^n}+\frac{P_{z0}^n\hat{z}-\mathbf{P^n}}{\{T_{1n},T_{2n},T_{2n}\}},\end{aligned} \tag{1}$$

where Q is the slowing-down factor, $\mathbf{P^e}$ and $\mathbf{P^n}$ are the spin polarizations of alkali electron and ^{21}Ne nucleus, respectively. $\mathbf{B}$ and $\mathbf{\Omega}$ are external magnetic field and angular frequency of the mechanical rotation. The Fermi-contact interaction between alkali atoms and ^{21}Ne atoms can be described by an effective magnetic field $\lambda M_0^{\rm e,n}\mathbf{P^{e,n}}$, where M_0^n (M_0^e) is the maximum magnetization of ^{21}Ne nucleon (alkali electron) [2], where $\lambda = 8\pi\kappa_0/3$, where κ_0 is the enhancement factor. $T_{1e}\equiv 2\pi/R_1^e$ and $T_{2e}\equiv 2\pi/R_2^e$ are the longitudinal and transverse relaxation rates for alkali electron spin, respectively, and $T_{1n}\equiv 2\pi/R_1^n$ and $T_{2n}\equiv 2\pi/R_2^n$ are the longudinal and transverse relaxation times for the ^{21}Ne nucleon spin. P_{z0}^e and P_{z0}^n are the equilibrium polarizations of alkali electron spins and noble-gas nuclear spins.

For spatially overlapping Rb and ^{21}Ne spins, each spin species experiences an effective magnetic field due to the magnetization of the other, which is enhanced by the Fermi-contact interactions. For spherically uniform polarized cell, the effective fields are

$$\begin{aligned}\tilde{\mathbf{B}}^{\mathbf{e}} &= \lambda M_0^e\mathbf{P^e} = \frac{8}{3}\pi\kappa_0 M_0^e\mathbf{P^e},\\ \tilde{\mathbf{B}}^{\mathbf{n}} &= \lambda M_0^n\mathbf{P^n} = \frac{8}{3}\pi\kappa_0 M_0^n\mathbf{P^n},\end{aligned} \tag{2}$$

where κ_0 is the enhancement factor due to Fermi-contact interaction. In our experiment, the coupled spin ensembles are polarized along the $\hat{z}$ axis. Hence the effective fields are mainly along the $\hat{z}$ axis.

The response to quasi-static signals, such as magnetic field $B_{y/x}$, inertial rotation Ω_y and exotic fields $b_y^{\rm Ne/e}$ is

$$P_x^e = \frac{P_z^e\gamma_e}{R_2^e}\left(b_y^{\rm Ne}-b_y^e+\frac{\Omega_y}{\gamma_{\rm Ne}}+\frac{\delta B_z}{\tilde{B}_z^c}B_y+\frac{\gamma_e\delta B_z^2}{R_2^e\tilde{B}_z^c}B_x\right), \tag{3}$$

where $\delta B_z = B_z-\tilde{B}_z^c$ is the deviation of bias field B_z from the self-compensation point $\tilde{B}_z^c=\tilde{B}_z^n+\tilde{B}_z^e$. When setting $\delta B_z=0$, the responses to magnetic fields B_y and B_x are suppressed, which is known as the SC regime. In this regime, the comagnetometer is no longer disturbed by low-frequency magnetic noise, which is the dominate noise in many spin-based precision measurements. In addition, the alkali atoms experience a small net field of $B_z+\tilde{B}_z^n=-\tilde{B}_z^e$, leading to the alkali atoms working in the SERF regime. Hence the comagnetometer retains ultrahigh sensitivity to non-magnetic fields Ω_y and $b_y^{\rm Ne/e}$.

In the SC regime, the response to cosine oscillation signals is given by

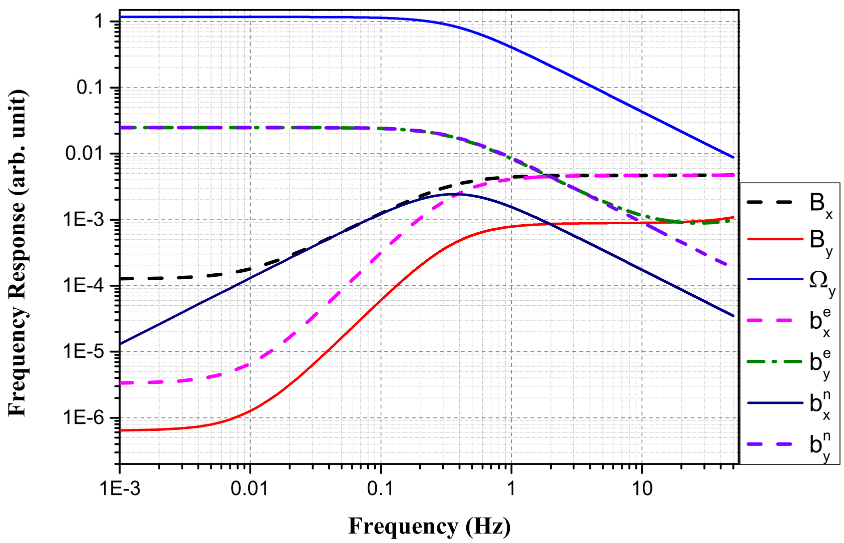

Supplementary Figure 3. Frequency responses to different signals. The frequency responses to magnetic fields $B_{x/y}$, inertial rotation Ω_y, exotic fields coupled to electron spins $b^e_{x/y}$, and exotic fields coupled to nuclear spins $b^{\rm Ne}_{x/y}$. The signals mainly coupled to nuclear spins ($b^{\rm Ne}_{x/y}$ and Ω_y) decrease with the frequency due to the narrow bandwidth of ^{21}Ne nuclear spins, i.e. small R^n_2. The responses of magnetic fields $B_{y/x}$ increase with frequency to the plateau due to the interactions between electron spins and nuclear spins cannot suppress the magnetic noises with high frequency. The frequency responses of b^e_x and b^e_y are similar to the frequency responses of SERF magnetometer with a bias field $\tilde{B}^e_z$ along the $\hat{z}$ axis.

$$
\begin{aligned}
P^e_x &= \mathrm{Re}(P^e_\perp) = \mathrm{Re}(P^e_1 e^{i\omega t} + P^e_2 e^{-i\omega t}),\\
P^e_1 &= \frac{P^e_{z0}\gamma_e}{2}\frac{(\omega - iR^n_2)B_\perp + (iR^n_2 Q/\gamma_e + \omega^n_z/\gamma_{eff})\Omega_\perp + (-iR^n_2 + \omega^n_z + \omega)b^e_\perp - \omega^n_z b^{\rm Ne}_\perp}{R^e_2(i\omega + R^n_2 + i\omega^n_z) + \omega^e_z(\omega - iR^n_z)},\\
P^e_2 &= \frac{P^e_{z0}\gamma_e}{2}\frac{(-\omega - iR^n_2)B_\perp + (iR^n_2 Q/\gamma_e + \omega^n_z/\gamma_{eff})\Omega_\perp + (-iR^n_2 + \omega^n_z - \omega)b^e_\perp - \omega^n_z b^{\rm Ne}_\perp}{R^e_2(i\omega + R^n_2 + i\omega^n_z) + \omega^e_z(\omega - iR^n_z)},
\end{aligned} \tag{4}
$$

where $P^e_\perp = P^e_x + iP^e_y$, $\omega^e_z = \gamma_e\tilde{B}^e_z$ and $\omega^n_z = \gamma_n\tilde{B}^n_z$. ω is the frequency of signals. $B_\perp = B_x + iB_y$, $\Omega_\perp = \Omega_x + i\Omega_y$, $b^e_\perp = b^e_x + ib^e_y$, and $b^{\rm Ne}_\perp = b^{\rm Ne}_x + ib^{\rm Ne}_y$ are the amplitudes of the signals. $\gamma_{eff} = \gamma_e\gamma_n/(Q\gamma_n + \gamma_e)$ is the effective gyromagnetic ratio, which indicates the rotation signal is amplified by a factor $(Q\gamma_n + \gamma_e)/\gamma_n$ using ^{21}Ne-Rb comagnetometer instead of Rb magnetometer.

Based on the experiment conditions of this work, we compare the difference in the frequency responses to magnetic field $B_{y/x}$, inertial rotation Ω_y, exotic fields coupled to electron spins $b^e_{x/y}$, and exotic fields coupled to nuclear spins $b^{\rm Ne}_{x/y}$. As shown in the Supplementary Fig. 3, the signals mainly coupled to nuclear spins ($b^{\rm Ne}_{x/y}$ and Ω_y) decrease with the frequency due to the narrow bandwidth of ^{21}Ne nuclear spins, i.e. small R^n_2. The responses of magnetic fields $B_{y/x}$ increase with frequency to the plateau due to the interactions between electron spins and nuclear spins cannot suppress the magnetic noises with high frequency. The frequency responses of b^e_x and b^e_y are similar to the frequency responses of SERF magnetometer with a bias field $\tilde{B}^e_z$ along the $\hat{z}$ axis.

B. Energy sensitivity of the comagnetometer

The magnetometer has two types of spices, the alkali atoms and noble-gas atoms. The electron spins and nuclear spins in these atoms are all sensitive to exotic fields. The fraction of neutron spin in Rb nucleus are assumed to be zero based on the basic nuclear shell model, and the fraction of polarisation for proton spin is $\eta^{\rm Rb}_p = 0.29$ [3]. The exotic field directly sensed by

Rb proton spin is

$$\delta E_{p,\mathrm{Rb}} = \eta_{p,\mathrm{Rb}} \mu_{N,\mathrm{Rb}} b_y^p \tag{5}$$

The exotic field sensed by the Ne atoms will make Ne spins (magnetisation) rotate, and this magnetisation is then detected by Rb atoms. The energy shift from this effect is

$$\begin{aligned} \delta E_{n,\mathrm{Ne}} &= \eta_{p,\mathrm{Ne}} \mu_B \delta \tilde{B}_n \\ &\approx \eta_{p,\mathrm{Ne}} \mu_B \tilde{B}_n \frac{b_y^p}{B_c} \\ &\approx \eta_{p,\mathrm{Ne}} \mu_B b_y^p, \end{aligned} \tag{6}$$

where we assumed $\tilde{B}_n \approx B_c$ to first order, and $\eta_p^{\mathrm{Ne}} = 0.04$ [4, 5]. Because μ_B is three orders of magnitude larger than μ_n, comparing these two equations, we find that the indirect effect via neon is much larger. The coupling to Rb neutrons are assumed to be negligible because its neutron polarisation is zero. For the coupling to electron spins, the energy resolution is comparable to that of the recent work in the Supplementary Ref. [6] and is thus not compared in our manuscript.

Supplementary Note 3. DATA ANALYSIS

In this section we discuss the data analysis procedure and the uncertainties of the final result. In subsection A, we discuss how the data are processed and characterize the source of uncertainties. In subsection B, C, D, the statistical and systematic uncertainties be discussed. Concerning the latter, for a search experiment, one should distinguish the factors that affect the detection power (e.g., spurious sources of the signal) and calibration errors. Calibration errors, if they are relatively small, have small effect on the discovery potential, however become important if the effect is observed and its characteristics need to be measured precisely. While we have not observed the exotic effect in this work, we have carried out an analysis of systematic uncertainties in the calibration, as presented below.

A. Data Taking and Analysis Procedure

The data were taken in 26 runs. Before each run, a field-zeroing procedure is executed to compensate the possible drift of the system and the calibration procedure is executed to obtain the calibration factor $\kappa_n(0.84\,\mathrm{Hz})$.

1) The signals of the comagnetometer and the rotation angle of the W-Al ring, which is recorded by an encoder, are collected simultaneously. The angular velocity of the rotation is obtained by the derivative of the rotation angle.

2) The raw data S_{raw} of the comagnetometer is phase-shifted based on the phase response of the comagnetometer to match the exotic field and the response signal, as shown in Fig. 2. For our 0.84 Hz signal, the time delay is Δt, as characterized in subsection D. The data are shifted by Δt forward and are denoted as S_{shift}.

3) As shown in Fig. 2 c in the main text, the data are cut into segments based on the phase of the rotation, and the length of each segment is $T/2$, i.e. half period of the modulation. Then the mean value of each segment is derived and noted as $\bar{S}_{2i,+}^{\exp}$ and $\bar{S}_{2i,-}^{\exp}$ for the positive and negative part respectively.

4) Assuming that the background signal is time-dependent, i.e. $n(t) = a + b*t + c*t^2$, where a, b and c are constants, the DC bias field and low-frequency drifts can be removed by evaluating

$$\bar{S}_i = \frac{1}{8}\left[\bar{S}_{2i,+} - 3\bar{S}_{2i,-} + 3\bar{S}_{2i+1,+} - \bar{S}_{2i+1,-}\right]. \tag{7}$$

The $\bar{S}_i$ is the effective amplitude of the signal for the i-th period of data.

5) The $\bar{S}_i^{\exp}$ is turned into the $\bar{b}_i^{\exp}$ using calibration factor $\kappa_n(0.84\mathrm{Hz})$.

6) Simulate the exotic field b^{sim} using Eq. (3) by inputting angular velocity, distances, nucleon density, etc. The nucleon distribution in the mass source is simulated with Monte Carlo method.

7) Find the effective magnitude of $\bar{b}_i^{\mathrm{sim}}$ using the method in step 4).

8) Find the coupling constant using

$$f_i = \frac{\bar{b}^{\exp_\mathrm{i}}}{\bar{b}^{\mathrm{sim}}}. \tag{8}$$

The statistical result $f = \bar{f} \pm \delta f_{\mathrm{stat}}$ can be obtained by analyzing all the periods.

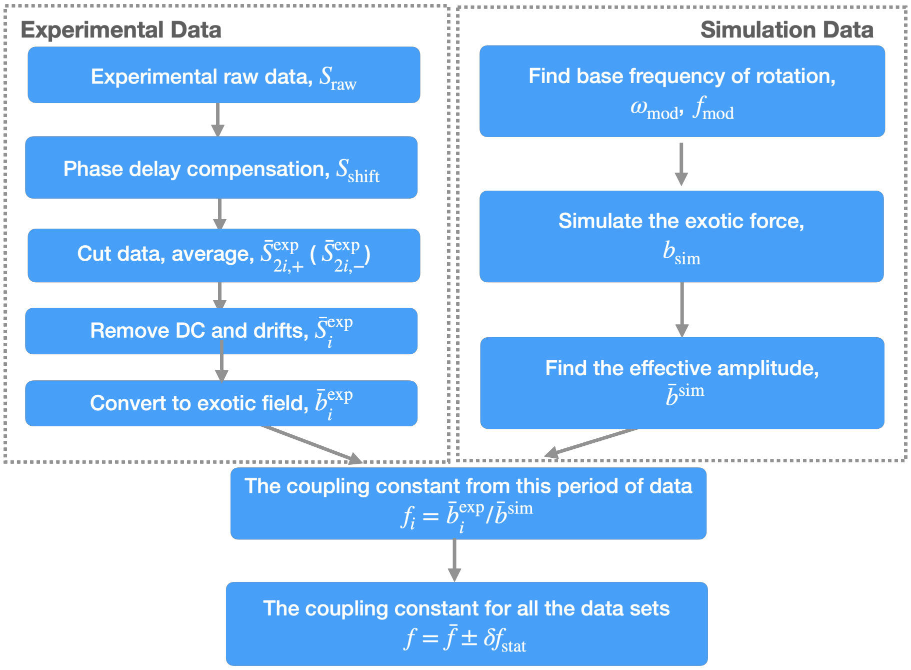

Supplementary Figure 4. The procedures of data analysis

As shown in the Supplementary Eq. 8, the coupling constant is a function of the experimental data and simulation data, thus the uncertainties also come from those sources. For the experimental data, there are two types of noise: 1) the statistical uncertainty, which comes from the spurious signals, such as magnetic noise and vibration noise. 2) the systematic uncertainty, which comes from the mass source and the detector. The uncertainty of the mass source causes the systematic uncertainty in simulating the $\bar{b}_i^{sim}$, including the uncertainties of parameters such as nucleon number from the mass source, the distance between the source and the sensor, and the velocity of the tungsten source. The systematic uncertainty from the detector is mainly due to the uncertainty of the calibration factor κ_n. These uncertainties are discussed in the following.

B. Statistical Uncertainty

Considering all the data, the statistical uncertainty is $\delta b^{\rm Ne} = 7.1$ aT. There are many kinds of noise causing statistical uncertainties, among which the two major sources are the magnetic noise, and the vibration noise. We analyse these below.

1. *Magnetic Noise Analysis*

The Sensitivities of searching for dark matter and exotic fields based on atomic spins are often limited by the magnetic noise. In this experiment, the major magnetic noise may come from 3 sources, (1) the environment, (2) the magnetic shield itself, (3) the electric heater and the other objects inside the magnetic shield.

(1) Environment magnetic noise. In order to suppress the environment magnetic noise, for example the geomagnetic field, we use multi-layer magnetic shields. The magnetic shields consist of five layers of high permeability permalloy (μ-metal) and a low-noise innermost Mn-Zn ferrite. The magnetic shielding factor is measured using a large three-axis coils and a small high-sensitivity commercial atomic magnetometer (QuSpin), as shown in the Supplementary Fig. 5 (a). The coil, which is powered by a precise current source, generates a uniform magnetic field B_{ext} up to 6×10^5 nT in the center. The commercial atomic magnetometer with a sensitivity on the order of 10 fT/hz$^{1/2}$ is used to measure the residual magnetic field B_{int} inside the shield. The measured radial shielding factor B_{ext}/B_{int} is shown in the Supplementary Fig. 5 (b). The shielding factor of the 5-layer permalloy shield and ferrite shield is better than that of the 5-layer permalloy shield, while the shielding factor decreases with frequency. This is probably due to the low conductivity of Mn-Zn ferrite. The shield factor at the operation frequency 0.84 Hz is about 2×10^8.

(2) Magnetic noise from the shielding material. Typically, although the permalloy shield has a good shielding factor, it also

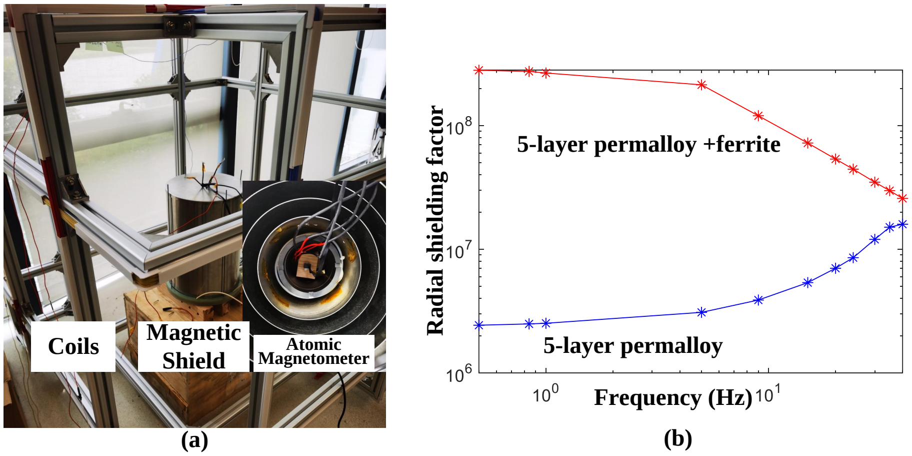

Supplementary Figure 5. The geometry of the magnetic shield and the shielding factor. (a) The magnetic shield consists of five layers of μ-metal shield and a low-noise innermost ferrite shield. A set of three-axis coils is used to generate external magnetic field B_{ext} to calibrate the shielding factor. A QuSpin magnetometer is used to measure the suppressed magnetic field B_{ins} inside the magnetic shield. (b) The measured magnetic shielding factors B_{ext}/B_{int} as a function of frequency. The red dots are the shielding factors of the five-layer permalloy shield together with the ferrite shield. The blue dots are that of the five-layer permalloy shield only.

generates magnetic noise due to the thermal magnetization noise and Johnson current noise. In order to suppress the magnetic noise of the magnetic shield, we use the low-noise Mn-Zn ferrite shield for the innermost shielding layer. The Mn-Zn ferrite has a low conductivity, leading to a low Johnson current noise. In this experiment, different kinds of Mn-Zn ferrite with different relative permeability and geometry were tested. After comparing, we chose the one whose real and imaginary components of the relative permeability were measured to be $\mu'/\mu_0 = 6308$ and $\mu''/\mu_0 = 45$, respectively. The length, inner diameter and thickness of the cylindrical ferrite shield are 22 cm, 11.4 cm and 1.3 cm, respectively. Although the AC electric heater is enclosed inside a vacuum chamber whose outer surface is wrapped with water-cooled tube, the actual temperature of the inner surface of the ferrite shield is still higher than room temperature and is measured to be 47°C. Using the above parameters, the magnetic noise of the ferrite shield is calculated to be $2.5f^{-1/2}$ fT [1] (f is the frequency in Hz), i.e. 2.8 fT/Hz$^{1/2}$ at the modulation frequency of the nucleon source (0.84 Hz). Since the comagnetometer is operated in the self-compensation regime, this further suppresses the magnetic noise (see the response curve in Fig. (7). The magnetic noise from the shielding is about 1.5 fT/Hz$^{1/2}$.

(3) Magnetic noise from the heater coils. In order to suppress the magnetic noise related to the electric heater, we modulate the current to 200 kHz AC current, far away from the modulation frequency of the exotic field b_y^n. The two heater coils are printed in one flexible board but with different directions of current flow. Hence the magnetic fields of the two coils compensate each other. To avoid magnetic noise of the nearby objects, we use the nonmagnetic plastic (PEEK) to build the vacuum chamber and the holder of the oven. We avoid using metal material inside the shield except for the alkali metal attached on the cell wall, the copper of the heater coil and magnetic field coils. The magnetic noise from the alkali metal and the copper are evaluated to be on the order of aT/Hz$^{1/2}$. The fluctuations of the currents in the coils is also very small.

2. *Vibration Analysis*

We measure the vibrations of the ground and the comagnetometer rack with a high precision seismometer (CMG-3ESP, Guralp). As shown in the Supplementary Fig. 6 (a), the vibration noise spectrum of the ground is below than 1×10^{-7}m/s^2/Hz$^{1/2}$ at the modulation frequency 0.84 Hz of the exotic signal. The vibration noise increases from 1 Hz and reaches a plateau around 3 Hz. In the vibration noise spectrum of the comagnetometer rack, there are many peaks around 1.5 Hz, 3.8 Hz, 5.2 Hz and so on.This peaks are due to the resonance vibration of the rack amplifies the vibrations of the cooling fans of various instruments and mechanical pump on the rack. Higher frequency peaks in the spectrum are higher order harmonics. The operation frequency 0.84 Hz is chosen to avoid the vibration resonance frequencies. There are some kind of beating pattern in Figure 2 in the main text, which are due to the resonance vibration. We also verify that this pattern is not related to the modulated exotic field by completely stop the power supply of motor and other things related to exotic field.

Although we try to suppress the vibration noises, the vibration can still manifest as a form of spurious signal in some conditions. As shown in the Supplementary Fig. 6 (b), we compare two extreme conditions, where condition 1 is at night with quite environment, while condition 2 is at the noisy moment in the daytime, when machines and air conditions are turned on in nearby laboratories and vehicles and people pass by the laboratory. The vibration noise would be 3 to 6 times worse at low frequency.

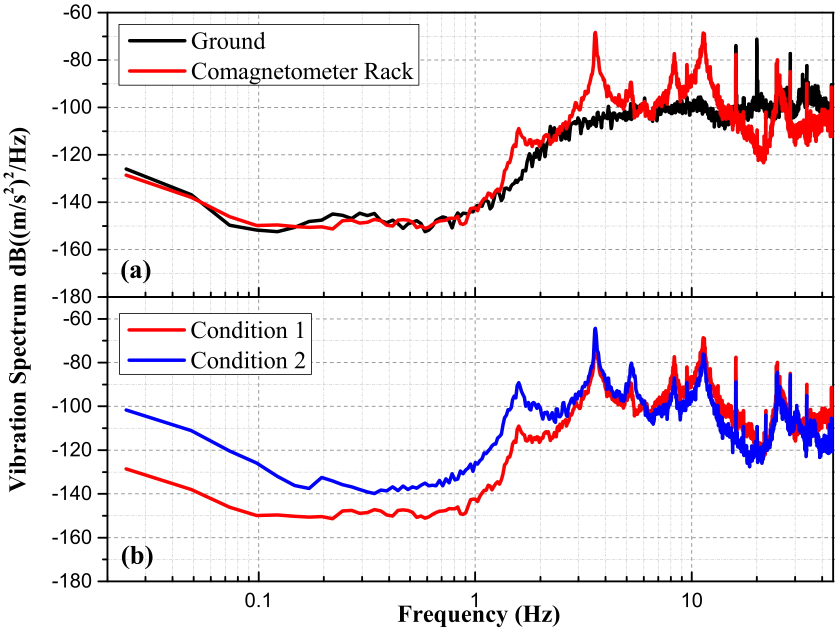

Supplementary Figure 6. Vibration noises. (a) The comparison of the vibrations of the ground and the comagnetometer rack under the same condition. There are many peaks in the spectrum of the comagnetometer rack, which are due to resonant vibrations of the rack amplifying the vibrations of the cooling fans of various instruments and mechanical pump on the rack. (b) Comparison of the vibrations of the comagnetometer rack under two extreme conditions. Condition 1 is at night with quiet environment, while condition 2 is during daytime with noise from machines and air conditioners in nearby laboratories as well as people walking in and outside the laboratory.

This might be the reason of error bar differences in Fig. 3. Besides, the environment temperature fluctuation and air convection would also cause fluctuations of error bars.

C. Systematic Uncertainty from the Mass Source

1. *Interferance between the source and the sensor*

(1) The magnetic leakage from the W-Al ring and the motor is measured with a fluxgate magnetometer. At the position near the magnetic shielding of the comagnetometer, the magnetic leakage is smaller than 0.1 nT around 0.84 Hz. After the decay with the distance and being shielded by five layers of mu-metal shield and one layer of ferrite shield, this field is estimated to be less than 1 aT and therefore is negligible.

(2) Vibrational coupling of the nucleon source is reduced by optimizing the parameters in the servo motor, and further isolated by placing the test mass and the comagnetometer on separate foundations and additionally to mount the comagnetometer on a vibration-isolation platform. An aluminum enclosure around the nucleon source is used to shield acoustic coupling through the air.

(3) Efforts are made to eliminate the possible electromagnetic cross-talk between the comagnetometer and the motor. The comagnetometer is installed in a magnetic shielding room (about $10 \times 4 \times 3\,\mathrm{m}^3$), while the servo motor is controlled with a separate computer and the control units are placed outside the shielding room (20 m). The connection cable is shielded from radio-frequency noise. In addition, they are powered from different supplies. The comagnetometer is powered by an uninterrupted power supply (UPS) with a power purifier in the circuit.

The isolation techniques are proved to be successful. We don't find any cross-talk between the source and the comagnetometer system.

2. *Error Propagation of the Mass Source*

As shown in Eq. (3) in the main text, the exotic force is a function of the velocity $\mathbf{v}$, the distance r, and the direction $\hat{r}$, and the nucleon distribution in the test material. The experimental parameters, such as rotation angular velocity ω, the W-Al ring radius

R, position and mass can be converted to the variables in Eqs. (3) and (4) in the main text, and thus their contribution to the statistical uncertainty of f can be obtained by a standard error-propagation method. Most of these parameters are independent thus their contribution to the error budget add in quadrature. Some parameters are correlated, for instance, the modulation frequency f, the phase shift ϕ, and calibration factor κ_n, since the phase shift and κ_n are frequency-dependent. The frequency uncertainty can contribute an uncertainty to κ_n on the order or less than 0.01%, corresponding to an uncertainty of $\delta_f^{\kappa} \ll 0.001$. The phase shift uncertainty will affect the $\bar{b}^{sim}$ by a factor less than 10^{-2}, corresponding to a the coupling constant of $\delta_f^{\phi} < 0.001$. Their error contribution to f are estimated by $\delta_f = \delta_f^{\kappa} + \delta_f^{\phi} < 0.001$. Please note that this δ_f^{κ} is many orders of magnitude less than the uncertainty of κ_n determined by the experimental parameters, see section D. Compared to the statistical uncertainty, most contributions to the systematic uncertainty are small, thus the correlation of parameters does not significantly affect the final uncertainty.

D. Analysis of the systematic error of the comagnetometer

The stability of the SERF comagnetometer is vital for long-term measurement. Typically, the drifts of the comagnetometer signal can be divided into two types. One is the drift of the calibration factor κ_n, which is due to the drift of the operation conditions of the comagnetometer. The other is the drift of the interference signals, such as magnetic fields. In the measurement of the exotic field b_y^{Ne}, the drift of the calibration factor κ_n would cause a systematic error, while the signal drift and noise would interfere with the b_y^{Ne}, which contributes to the statistical error. In order to avoid the drift of the interference signals, we modulate the b_y^{Ne} at about 0.84 Hz to remove the low-frequency drift of the spurious signal, see subsection A.

In order to characterize the comagnetometer systematic error, on the one hand, we establish a response model of the comagnetometer and evaluate the systematic error from the drifts of the operation conditions, such as the drifts of the pump-laser intensity, frequency and alignment, etc. On the other hand, we directly measure the long-term stability of κ_n as discussed below.

(1) Response model of comagnetometer.

In this experiment, we modulate the exotic signal b_y^{Ne} at about 0.84 Hz. Considering the frequency response, the $\kappa_n(\mathrm{DC})$ and the $\kappa_n(0.84\mathrm{Hz})$ are different, there is a correction factor $\eta_{b_y^n}$ between them. According to the Supplementary Eq. 4, the frequency response to $b_y^n cos(\omega t)$ is given by

$$S_x^e(b_y^{\mathrm{Ne}}) = \kappa_n(0.84\mathrm{Hz})\mathrm{b_y^{Ne}}\cos(\omega\mathrm{t}+\phi_{\mathrm{b_y^n}}) = \kappa_\mathrm{n}(\mathrm{DC})\eta_{\mathrm{b_y^n}}\mathrm{b_y^{Ne}}\cos(\omega\mathrm{t}+\phi_{\mathrm{b_y^n}}), \tag{9}$$

where $\kappa_n(\mathrm{DC}) = \mathrm{K_1K_2K_3}$ is the scale factor for quasi-static b_y^{Ne}. The correction factor $\eta_{b_y^n}$ and the phase delay $\phi_{b_y^n}$ determine the frequency response to b_y^{Ne}. K_1 is the factor to turn b_y^{Ne} into the transverse Rb spin polarization P_x^e, K_2 is the factor to turn P_x^e into optical rotation θ, K_3 is the factor to turn θ into the output voltage signal S_x^e [7],

$$\begin{aligned} K_1 &= \frac{\gamma_e P_z^e}{R_2^e}, \\ K_2 &= \frac{1}{2} l n_e r_e c \frac{f_{D1}^{\mathrm{Rb}}(\nu_{\mathrm{pr}} - \nu_{D1}^{\mathrm{Rb}})}{(\nu_{\mathrm{pr}} - \nu_{D1}^{\mathrm{Rb}})^2 + (\Gamma_{D1}^{\mathrm{Rb}}/2)^2}, \\ K_3 &= \zeta_{\mathrm{PD}} \alpha_{\mathrm{PEM}} I_{\mathrm{pr0}} \exp\left[-\frac{n_e l r_e c f_{D1}^{\mathrm{Rb}} \Gamma_{D1}^{\mathrm{Rb}}/2}{(\nu_{\mathrm{pr}} - \nu_{D1}^{\mathrm{Rb}})^2 + \left(\Gamma_{D1}^{\mathrm{Rb}}/2\right)^2} \right], \end{aligned} \tag{10}$$

where l is the diameter of the cell, n_e is the number density of Rb, r_e is the classical radius of electron, c is the speed of light, f_{D1}^{Rb} is the oscillator strength, ν_{D1}^{Rb} is the resonance frequency of Rb D1 line, $\Gamma_{D1}^{\mathrm{Rb}}$ is the pressure broadened linewidth. ζ_{PD} is the photoelectric conversion efficiency of the PD. α_{PEM} is modulation amplitude of the PEM. I_{pr0} is the incident intensity of the probe light.

The correction factor $\eta_{b_y^n}$ and the phase delay $\phi_{b_y^n}$ are given by

$$\begin{aligned} \eta_{b_y^n} &= \sqrt{\left[\mathrm{Im}(P_1^{b_y^n} + P_2^{b_y^n})\right]^2 + \left[\mathrm{Re}(P_1^{b_y^n} - P_2^{b_y^n})\right]^2}, \\ \phi_{b_y^n} &= \arccos\left[\frac{\mathrm{Im}(P_1^{b_y^n} + P_2^{b_y^n})}{\eta_{b_y^n}}\right]. \end{aligned} \tag{11}$$

where $P_1^{b_y^n}$ and $P_2^{b_y^n}$ are from the Supplementary Eq.4. $\eta_{b_y^n}$ and $\phi_{b_y^n}$ are characterized by four systematic parameters $\tilde{B}_z^e$, $\tilde{B}_z^n$, R_2^e, and R_2^n.

(2) Systematic error of κ_n(DC).

The κ_n(DC) is mainly affected by the drifts of experimental conditions: cell temperature δT_c, pump-light intensity δI_{pu}, frequency $\delta \nu_{pu}$, position δP_{pu}, and polarization δs_{pu} as well as probe-light intensity δI_{pr}, frequency $\delta \nu_{pr}$, and position δP_{pr}.

Estimation. We can calculate the systematic error of κ_n(DC) due to the drifts of experimental conditions based on the Supplementary Eq. 10. The 4-hr drifts of experimental conditions are summarized in the Supplementary Table in 1. The drifts δT_c, δI_{pu}, $\delta \nu_{pu}$, δI_{pr}, $\delta \nu_{pr}$, δs_{pu} are measured results. While we did not measure the light-beam motion directly, we use the values in the Supplementary Ref. [8] to estimate the drifts in κ_n(DC). We assume these drifts are independent, so that the change of κ_n(DC) is the quadrature sum of each drift of about 0.3 %.

Check. We directly measure the stability of the calibration factor κ_n(DC) on a longer time scale (the Supplementary Fig. 7). The value of κ_n(DC) is stable over the 4-hr measurement duration with a standard deviation about 0.6 %. Therefore, we choose four hours as the measurement period based on the stability of the system and convenience. The measured uncertainty is slightly larger than the calculated one, which is probably due to the estimated light-beam motion being smaller than actual value and the fact that we have not accounted for the change in lab temperature.

To ensure the stability of operation, the main experimental conditions, including the pump-light intensity and frequency, the cell temperature, and probe-light intensity are feedback controlled and monitored, such that we can check the status of the comagnetometer during operation without disturbing the measurement to make full use of the measurement time.

(3) Systematic errors in $\eta_{b_y^n}$ and $\phi_{b_y^n}$.

The four parameters ($\tilde{B}_z^e$, $\tilde{B}_z^n$, R_2^e, and R_2^n) determining $\eta_{b_y^n}$ and $\phi_{b_y^n}$, are mainly affected by the drifts of T_c, I_{pu}, ν_{pu}, P_{pu}, and s_{pu}.

Estimation. We calculate the corresponding drifts of the four systematic parameters using the measured δT_c, δI_{pu}, $\delta \nu_{pu}$, δs_{pu} [9]. We assume these drifts are independent. Thus effects of these drifts are added in quadrature. The corresponding drifts of $\tilde{B}_z^e$, $\tilde{B}_z^n$, R_2^e, and R_2^n, which are about 0.08% (110.6 nT), 0.08 % (579.4 nT), 0.05 % (3715 1/s), $<$ 0.01% (0.063 1/s) respectively. Based on the Supplementary Eq. 11, the total influences on the $\eta_{b_y^n}$ and the $\phi_{b_y^n}$ are calculated to be 0.3 % and 0.1 % respectively, see Supplementary Table in 1.

Check. We directly measure the fluctuations of the relevant parameters as a function of the measurement length. We find the influence of R_2^n on the correction factor $\eta_{b_y^n}$ is the least out of those for the four relevant parameters. Based on Eq. 11, ignoring R_2^n only changes the $\eta_{b_y^n}$ by 0.4% and the $\phi_{b_y^n}$ by 0.9%. Besides, $\tilde{B}_z^e$ and $\tilde{B}_z^n$ are all proportional to P_{z0}^e, $\tilde{B}_z^n = \zeta^{en}\tilde{B}_z^e$. The scaling factor ζ^{en} is almost constant and only slightly depends on temperature. Measurement of B_z^c can be used for monitoring of $\tilde{B}_z^e$ and $\tilde{B}_z^n$. The fluctuations of $\tilde{B}_z^c$ and R_2^e with different time scales are shown in the Supplementary Fig. 7. We measure the $\tilde{B}_z^c$ and R_2^e every 15 minutes for four hours to present the stability of the parameters during the 4-hr measurement duration. The $\tilde{B}_z^c$ and R_2^e are stable, about 690.2(2) nT and 3.93(17) $\times 10^3$ 1/s, respectively, within the 4-hr duration. In the Supplementary Fig. 7, we also show the parameters measured with a longer time scale to present the stability over one day. The fluctuations of $\tilde{B}_z^c$ and R_2^e are also small.

The typical operation procedure is that before every 4-hr measurement, we check and compensate small drifts of transverse magnetic fields $B_{x/y}$ and the longitudinal field $\tilde{B}_z^c$ [9]. Then we measure the scale factor κ_n(DC), $\tilde{B}_z^e$, $\tilde{B}_z^n$ and R_2^e, which are used to get the calibration factor $\kappa_n(0.84\,\text{Hz})$ as well as its uncertainty $\delta\kappa_n$ based on the Supplementary Eq. 9. Subsequently, we simultaneously measure the 4-hr signals of comagnetometer and the rotation of W-Al ring. And then we check the $\kappa_n(0.84\,\text{Hz})$ after the 4-hr data collection to ensure the stability of $\kappa_n(0.84\,\text{Hz})$. The overall calibration factor for the all 26 sets of 4-hr data is $\kappa_n(0.84\,\text{Hz}) = 1.67(5)\times 10^{-6}\,\text{V/fT}$. This overall calibration factor is used in the evaluation of the total systematic error in Supplementary Table 1.

Supplementary Table 1. The analysis of systematic error for the calibration factor.

Parameter	Value	$\delta\kappa_n$(DC)	$\eta_{b_y^n}$	$\phi_{b_y^n}$
Pump light intensity I_{pu}	0.1%	0.03%	0.2%	0.05%
Pump light frequency ν_{pu}	0.000001%(5 MHz)	0.000003%	0.00002%	0.000006%
probe light intensity I_{pr}	0.1%	0.1%	–	–
probe light frequency ν_{pr}	0.00001%(50 MHz)	0.005%	–	–
Cell temperature T_c	0.005%(0.01 °C)	0.02%	0.1%	0.03%
Pump light polarization s_{pu}	0.1%	0.03%	0.2%	0.05%
Pump beam position P_{pu} (estimated)	–	0.2%	–	–
Probe beam position P_{pr} (estimated)	–	0.2%	–	–
Final		0.3%	0.3%	0.08%
Final $\delta\kappa_n$(0.84Hz)				0.4%

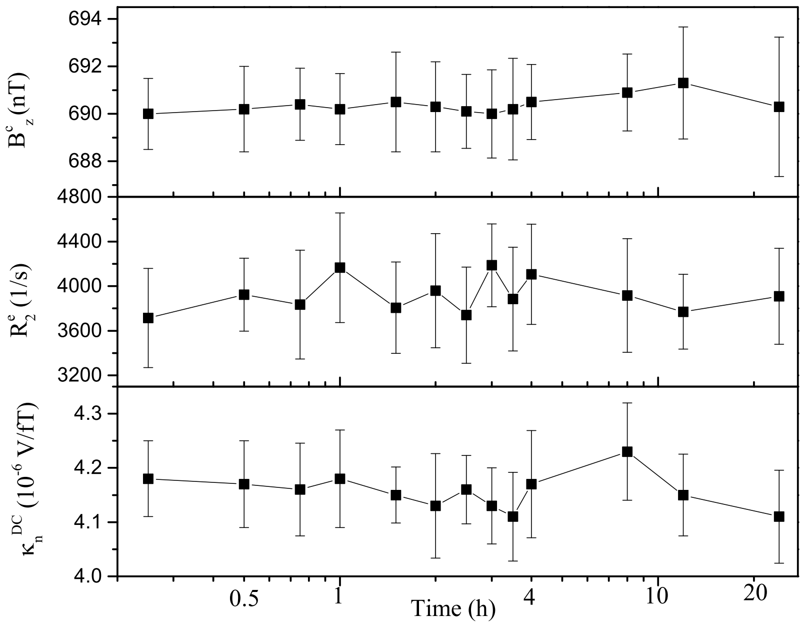

Supplementary Figure 7. Parameters stability. Measurement of main parameters $\tilde{B}^c_z$ and R^e_2, and the calibration factor κ_n(DC) for different time scale. Error bars represent statistical error.

Supplementary References

[1] T. Kornack, S. Smullin, S.-K. Lee, and M. Romalis, A low-noise ferrite magnetic shield, Applied physics letters **90**, 223501 (2007).

[2] M. Romalis and G. Cates, Accurate 3 he polarimetry using the rb zeeman frequency shift due to the rb- 3 he spin-exchange collisions, Phys. Rev. A **58**, 3004 (1998).

[3] D. J. Kimball, Nuclear spin content and constraints on exotic spin-dependent couplings, New J. of Phys. **17**, 073008 (2015).

[4] A. Almasi, J. Lee, H. Winarto, M. Smiciklas, and M. V. Romalis, New limits on anomalous spin-spin interactions, Phys. Rev. Lett. **125**, 201802 (2020).

[5] B. Brown, G. Bertsch, L. Robledo, M. V. Romalis, and V. Zelevinsky, Nuclear matrix elements for tests of local lorentz invariance violation, Phys. Rev. Lett. **119**, 192504 (2017).

[6] K. Wu, S. Chen, G. Sun, S. Peng, M. Peng, and H. Yan, New experimental limits on exotic spin-and velocity-dependent interactions using rotationally modulated source-masses and an atomic-magnetometer array, arXiv preprint arXiv:2109.13847 (2021).

[7] S. J. Seltzer, *Developments in alkali-metal atomic magnetometry* (Princeton University, 2008).

[8] J. Lee, A. Almasi, and M. Romalis, Improved limits on spin-mass interactions, Phys. Rev. Lett. **120**, 161801 (2018).

[9] T. W. Kornack, *A test of CPT and Lorentz symmetry using a potassium-helium-3 co-magnetometer* (Princeton University, 2005).
